# Exploring new technologies for the future generation: exploration–exploitation trade-off in an intergenerational framework

**DOI:** 10.1098/rsos.231108

**Published:** 2024-05-01

**Authors:** Yo Nakawake, Yutaka Kobayashi

**Affiliations:** ^1^ School of Economics and Management, Kochi University of Technology, Kochi, 780-8515, Japan; ^2^ Centre for the Study of Social Cohesion, University of Oxford, Oxford, OX2 6PE, UK; ^3^ Research Institute for Future Design, Kochi University of Technology, Kochi, 780-8515, Japan

**Keywords:** cumulative cultural evolution, individual learning, social learning, virtual arrowhead, transmission-chain experiment

## Abstract

Decision making on exploring or exploiting technology was studied by means of a laboratory experiment with a two-generation framework. In this framework, the design of a virtual tool is transmitted from the first to second generation, and hence, the former can help the latter by frequently exploring better tool designs but at the cost of reduced opportunities to exploit the existing tool to increase its own benefits. We set two experimental conditions (‘repaid’ and ‘unrepaid’) as well as a control condition (asocial), in which the second generation is absent. In the ‘repaid’ experimental condition, participants received an extra payment proportional to the score gained by the second generation, such that they were monetarily incentivized to help the second generation. Such an incentive was not given in the ‘unrepaid’ condition. An analysis of a formal model and computer simulations predicted that rational participants should increase investment in exploration only in the repaid condition when compared with the asocial control. The prediction was confirmed by the results of the experiment. These findings together suggest that humans may not have a propensity to invest in costly exploration of new technologies solely to help future generations.

## 1. Introduction

Exploration of new technologies for future generations is one of the key agendas for the sustainable development of society. As the United Nations set ‘World Creativity and Innovation Day’ and positioned technological innovation as one of the Sustainable Development Goals, there is now growing interest in the topic [1,2]. Innovative technologies benefit individuals and society, providing better solutions to problems faced in multiple domains (e.g. foraging, agriculture and manufacturing; [3–5]). At the same time, investments in technological exploration may be associated with a loss of opportunities that could have been used to gain immediate returns from exploiting existing technologies (e.g. hunting with known skills or manufacturing products with available technologies; [6–8]). The allocation of investment by an individual between exploration and exploitation in the presence of the trade-off should depend on whether only the benefit to the current generation is concerned or whether benefits to future generations, who will benefit from technologies culturally transmitted from the current generation, are also taken into account [9,10]. Specifically, one should invest more in exploration at the expense of myopic returns if benefits to future generations are concerned than if not.

Thus, the exploration–exploitation trade-off in culturally transmitted technology fundamentally involves the problem of cooperation with future generations or more generally with the successors of technology. Of course, innovative technologies contribute to the success of innovators themselves, but we are here concerned about whether the presence of successors results in an increased allocation of effort to exploration. Notably, the trade-off between exploration and exploitation differs from the problem of allocation to individual and social learning, to which quite a number of studies have been devoted, and which was also studied in terms of a social dilemma. This type of dilemma structure is widely known as the producer–scrounger game, where individual and social learners are regarded as the producers and scroungers of adaptive information, respectively [8,11,12]. Rogers’ paradox asserts that social learning (and hence the presence of culture) *per se* does not contribute to the fitness of a population, and the resolutions of the paradox are among the most notable results of this line of research [13–17].

Cooperation with future generations has been studied in terms of indirect reciprocity (i.e. pay-it-forward or upstream reciprocity; [18]) or common-pool resource games [19,20]. However, as revealed by behavioural experiments, cooperation with future generations cannot be robustly sustained [21] without institutional systems (e.g. democratic voting; [19]) or psychological treatments (e.g. inducing perspective-taking or emotional responses; [22,23]). Evolutionary analysis of pay-it-forward reciprocity (a chain of altruistic actions towards the third party) shows that it cannot evolve unless helping is returned to the helper with a certain probability [18]. In a similar vein, theory of cultural evolution shows that in the presence of the exploration–exploitation trade-off, the cumulative cultural evolution of technology is hardly possible unless technology is transmitted vertically from parents to offspring to prevent information free-riding [24]. This theoretical result suggests that, at least in the presence of the trade-off, investment in technology exploration cannot be promoted simply by the fact that technology is transmitted to someone, that is, the third party or non-kin [24]; some additional mechanisms, such as kinship or direct reciprocity, are required [18].

The goal of this article is to test the following two hypotheses: (i) exploration of technology is promoted solely by the fact that future generations inherit a form of technology; and (ii) it is promoted when individuals are additionally rewarded for providing increased benefits to the future generations. Note that the two hypotheses differ only in whether there is (hypothesis (ii)) or is not (hypothesis (i)) an extrinsic incentive to benefit future generations. Existing studies suggest that humans need extrinsic incentives to increase investment in technological exploration for future generations; thus, they predict that hypotheses (i) is false but hypothesis (ii) is true. We achieve the goal via three approaches: a formal model, computer simulations and a laboratory experiment, among which the experiment plays the central role and the other two supportive roles. We devote the remainder of this section to outlining these approaches (see §2–4 for details).

Note that while the above hypothesis (ii) is intuitive, it is unclear under what conditions and rewarding systems investment in exploration actually increases. The purpose of our first approach, a formal model, is to set out an appropriate reward system and to specify the conditions under which this reward system works as expected. We stress that this model is introduced primarily to justify the experimental design and to interpret the experimental results. Generalizing a number of specific aspects of the experiment, the model also serves to help extrapolate the results of the experiment. This model is *not* intended to describe cultural evolution in real populations and hence it is intentionally artificial and oversimplistic compared with mathematical models found in usual theoretical works of cultural evolution. For example, it intentionally excludes factors like environmental change, the loss of information during social transmission, the intragenerational transmission of technology and the intergenerational transmission of technology over three or more generations. In this model, we consider a sequential game of two players in which the technology accumulated by the first player (predecessor) is faithfully transmitted to the second player (successor). Each player faces the problem of optimally allocating time to exploration and exploitation. Exploitation is fundamental to the final pay-off. However, exploration is required for efficient exploitation. The reward system we propose here is designed to add a bonus to the first player’s pay-off, where the bonus is equal to the final pay-off to the second player. We call the set-up with this rewarding system the *repaid* condition as opposed to the *unrepaid* condition, in which there is no reward system. We show that the reward system of the repaid condition results in an increased investment in exploration by the first player under quite a broad range of conditions.

Although our formal model is developed to assist the experiment, the former ignored a number of non-ideal, disturbing features of the latter for analytical tractability. Therefore, it is not fully evident if rational players (in this article, we use the term ‘rational’ to mean the maximization of one’s own pay-off without regard to others’ pay-offs), which have proved to invest more in exploration in the repaid condition than in the unrepaid condition in the set-up of the formal model, would also do so in the laboratory experiment. Clearly, without this guarantee, neither a positive nor negative result of the laboratory experiment can be interpreted on the basis of the theory developed in the formal model. The purpose of our second approach, an *in silico* experiment or computer simulations, is to bridge this gap between the formal model and laboratory experiment. We assume that an artificial agent with a seemingly rational searching algorithm and an allocation strategy analogous to the optimal strategy found in the formal model undertakes the same task under the same conditions as in the laboratory experiment. We show that the agent invests more in exploration in the repaid condition than in the unrepaid condition, as predicted by the formal model.

Finally, the purpose of our third approach, a laboratory experiment, is to test both hypotheses (i) and (ii) using real participants. Although hypothesis (i) is evidently false from the standpoint of rational players, it is not trivial at all when applied to real humans [25]. The falsity of hypothesis (i) is worth challenging, especially in realistic conditions, which violate the idealized assumptions of the formal model, for example, when the performance of each technology is unknown before it is used, when information on performance is noisy, and when the exploration task itself is entertaining to some extent. The finding by previous studies that intergenerational cooperation generally needs institutions or emotional treatments [19,20,22] implies, on the other hand, that humans do have the potential to cooperate with the future generation, and various non-utilitarian factors inherent in real innovative practises, such as entertainment or sense of goal accomplishment, which are usually ignored in economic experiments, could elicit it. As an ideal experimental framework with these realistic features, we adopt the virtual arrowhead task [26,27], which has been successfully used to investigate multiple topics concerning cultural evolution [1,8,28–30]. For this task, participants design an arrowhead, whose performance depends on multiple attributes (e.g. length, width and depth). Participants do not know the performance of each arrowhead design before they go hunting with the designed arrowhead. Moreover, random noise is added to the performance scores of arrowhead designs gained through hunting. Such features allow us to evaluate the relevance of hypothesis (i) in controlled but comparatively realistic situations.

In the present experiment, we made two major modifications to the original arrowhead task to fit our purposes. Note that in the original task [26], participants design an arrowhead and then immediately go hunting in every trial, so there is no room for a time-allocation trade-off. The first modification we make is to introduce a trade-off structure by allowing participants to engage in only either exploration (designing) or exploitation (hunting) in each trial. The second major modification is to introduce intergenerational transmission as practised in many experiments using the transmission-chain method [29,31] and iterated learning [32,33]. Namely, participants are anonymously paired and serve as the first or second generation. The final arrowhead design of the first generation is used as the initial arrowhead design for the second generation. Based on the result of the formal model, we again set both the repaid and unrepaid conditions as well as the control for hypothesis testing, that is, the asocial condition, in which there is no intergenerational transmission. We test hypothesis (ii) (i.e. investment in exploration by the first generation is greater in the repaid condition than in the asocial condition) and hypothesis (i) (i.e. investment in exploration by the first generation is greater in the unrepaid condition than in the asocial condition).

The remainder of this article is structured as follows. In §2, we describe and analyse the formal model employed. In §3, we present the results of computer simulations. In §4, we describe the method (§4.1) and results (§4.2) of our laboratory experiment. In §5, we discuss the implications of the results obtained in §§2–4.

## 2. Formal model

Let us consider a sequential game of two players. A fixed amount T of time is available to each player, and the goal of a player is to maximize their own benefit by allocating time to two activities: exploration of novel technologies and exploitation of known technologies to extract benefits from the environment. For the first player, the efficiency of exploitation (or technology level) at any moment h∈[0,T] is assumed to be an increasing function f⁢(t) of the amount of time t<h that the player invested in exploration before time h . The first player gains a pay-off amount f⁢(t) by investing a unit amount of time in exploitation, given the current technology level f⁢(t) . The technology accumulated by the first player is faithfully transmitted to the second player such that the second player enjoys efficiency $f\,\left(t_{p}+t\right)$ at any moment h∈[0,T] , where *t*_*p*_ is the total amount of time invested by the first player in exploration and t<h is the amount of time invested by the second player in exploration before time h . For the arguments below, we assume not only that f⁢(t)≥0 and $f^{\prime }(t)>0$ but that log⁡f⁢(t) is concave, that is, $(\log f(t){)}^{\prime \prime }\leq 0$ , for any t∈[0,2⁢T] . This class of functions is broad enough to include all linear and concave increasing functions with non-negative codomains (functions satisfying f(t)≥0 , $f^{\prime }(t)>0$ and $f^{\prime \prime }\,\left(t\right)\leq 0$ ). Moreover, the class includes a class of convex functions such as $f\,\left(t\right)=e^{a\,t}$ ( a>0 ) or $f\,\left(t\right)={\left(a+t\right)}^{b}$ ( a≥0 , b>1 ). Note that f⁢(t) is analogous to the concept known as the ‘learning curve’ in behavioural science [34] and engineering management [35].

In theory, each player may fragment the time for exploration into several or many periods dispersed over timeframe [0,T] . However, given the deterministic and monotonic nature of function f , in any rational allocation strategy, exploitation should not precede exploration. Therefore, we may assume that the first player explores and accumulates technology up until time *t*_*p*_ and invests the remainder of time $T-t_{p}$ exclusively in exploitation. The same argument holds for the second player with *t*_*p*_ replaced by *t*_*s*_, the total investment in exploration by the second player. Given that the second player’s technology level is $f\,\left(t_{p}+t_{s}\right)$ at switching time *t*_*s*_, the total pay-off to the second player is given by:


(2.1) $$W_{s}=(T-t_{s})f(t_{p}+t_{s}).$$

For the first player’s pay-off, we consider two set-ups: *repaid* and *unrepaid* conditions. In the unrepaid condition, the first player’s pay-off $W_{p,u}$ (the first subscript stands for the ‘predecessor’) is determined by its own earnings alone, just as for the second player:


(2.2) $$W_{p,u}=\left(T-t_{p}\right)\,f\,\left(t_{p}\right).$$

However, in the repaid condition, an additional pay-off equivalent to the earnings of the second player is given as a bonus such that the pay-off to the first player is given by


(2.3) $$W_{p,r}=W_{p,u}+W_{s}=\left(T-t_{p}\right)\,f\,\left(t_{p}\right)+\left(T-t_{s}\right)\,f\,\left(t_{p}+t_{s}\right).$$

Of particular interest to us is whether the first player, if rational, explores technology for a longer time in the repaid condition than in the unrepaid condition. In the unrepaid condition, the first player’s pay-off $W_{p,u}$ is a function of its own choice *t*_*p*_ alone, and hence, the rational choice is simply the one that maximizes the single variable function $W_{p,u}$ . However, in the repaid condition, precise arguments require reference to game theory because the first player must consider the second player’s rational response to the first player’s choice. However, for our simple game, the first player’s rational choice can be easily derived through intuitive arguments without resorting to game theory, as described below. In the following, we focus on the main results of the optimal strategy for the first player, mostly omitting technical details. For completeness, in the electronic supplementary material, appendix S1, we provide a full analysis of the optimal strategies for both players with technical details (electronic supplementary material, appendix S1.1). Furthermore, in the electronic supplementary material, we also provide still more rigorous game-theoretical arguments, which do not rely on intuitive arguments, for theory-oriented readers (electronic supplementary material, appendix S1.2).

Note that in the repaid condition, there is no conflict of interest between the two players over the choice *t*_*s*_ of the second player, that is, the second player would choose *t*_*s*_ to maximize $W_{s}$ for a given *t*_*p*_. This choice must also be the one that maximizes $W_{p,r}$ for a given *t*_*p*_. Therefore, nothing changes even if we assume that the first player has control over both *t*_*p*_ and *t*_*s*_. Moreover, the basic part ( $W_{p,u}$ ) and the bonus ( $W_{s}$ ) have the same weights in the pay-off $W_{p,r}$ of the first player. Therefore, the situation for the first player, given that it has control over both *t*_*p*_ and *t*_*s*_, is essentially identical to the unrepaid condition with available time now being 2⁢T . Our problem hence reduces to the investigation of the effect of replacing T by 2⁢T in the pay-off function $W_{p,u}$ for the unrepaid condition. Note that $t_{p}=T$ or $t_{s}=0$ must hold for any rational combination of *t*_*p*_ and *t*_*s*_ because otherwise investment in exploration is fragmented into two separate periods from the viewpoint of the hypothetical first player endowed with an amount 2⁢T of available time, which is irrational.

Let us now focus on the optimization problem for the first player in the unrepaid condition, which should also give us the solution of the repaid condition according to the above arguments. For simplicity, here we assume that the optimal switching time $t_{p}^{*}$ for the first player, which maximizes $W_{p,u}$ in equation (2.2), is an interior point (i.e. $0<t_{p}<T$ ). Then, in the electronic supplementary material, appendix S1, we show that $t_{p}^{*}$ is the unique solution of the following equation:


(2.4) $$\frac{f^{\prime }\,\left(t_{p}\right)}{f\,\left(t_{p}\right)}=\frac{1}{T-t_{p}}.$$

**Figure 1.**
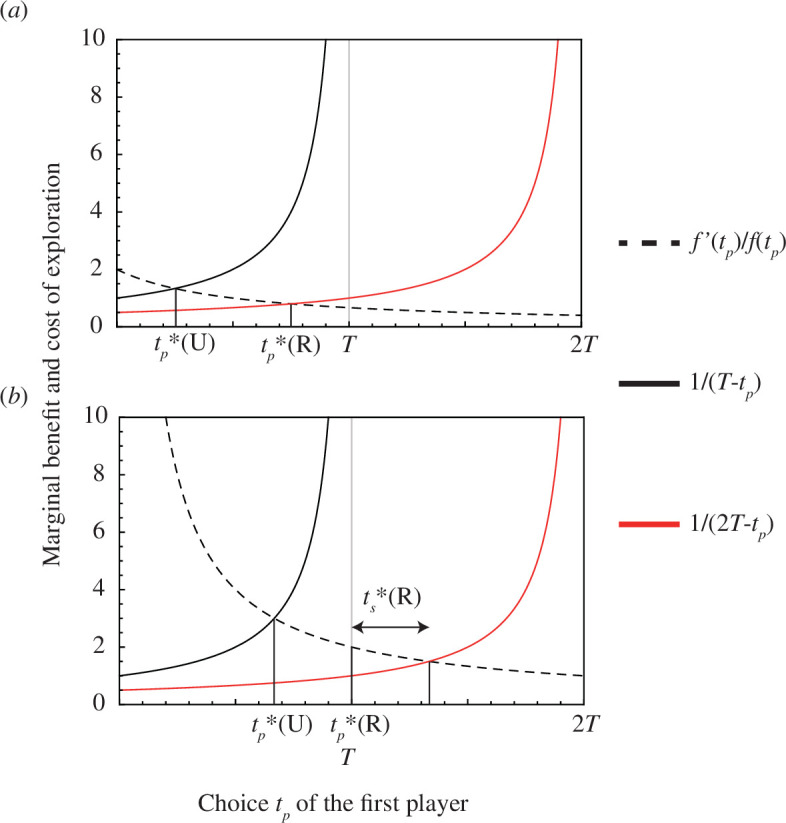
Rational choice for the first player in the unrepaid condition (marked by $t_{p}^{*}$ (**U**)) and the repaid condition (marked by $t_{p}^{*}$ (**R**)) derived from the formal model. The black dashed curve represents the marginal benefit of exploration $f^{\prime }\,\left(t_{p}\right)/f\,\left(t_{p}\right)$ . The black and red solid curves represent the marginal cost of exploration in the unrepaid condition $1/\left(T-t_{p}\right)$ and that in the repaid condition $1/\left(2\,T-t_{p}\right)$ , respectively. (*a*) The case of a linear function f⁢(t)=1+2⁢t . (*b*) The case of a convex function $f\,\left(t\right)=t^{2}$ . In panel (*a*), the rational choice for the second player in the repaid condition is $t_{s}^{*}=0$ . In panel (*b*), the rational choice for the second player in the repaid condition is indicated by the two-sided arrow and $t_{s}^{*}$ (**R**). The total time available to each player is T=1 .

In equation (2.4), the left-hand side (*l.h.s*.) and right-hand side (*r.h.s*.) represent the marginal benefit and cost, respectively, of increasing the investment *t*_*p*_ in exploration. For a full description of the solution including boundary cases, see the electronic supplementary material, appendix S1. Figure 1 illustrates the *l.h.s*. and *r.h.s*. of equation (2.4) as functions of *t*_*p*_ for a linear function f⁢(t)=1+2⁢t (figure 1*a*) and a convex function $f\,\left(t\right)=t^{2}$ (figure 1*b*). Given ${\left(\log f\right)}^{\prime \prime }\leq 0$ , the *l.h.s*. of equation (2.4) is a monotonically decreasing function of *t*_*p*_, while the *r.h.s*. is a strictly monotonic increasing function. The intersection of the two curves is the point where equation (2.4) holds true, the *x*-coordinate of which gives the optimal strategy $t_{p}^{*}$ for the first player in the unrepaid condition.

Now, the effect of replacing T by 2⁢T in the pay-off function is obvious from figure 1. The curve for $1/\left(2\,T-t_{p}\right)$ is lower than that for $1/\left(T-t_{p}\right)$ and hence $t_{p}^{*}$ should be greater in the repaid condition than in the unrepaid condition. Note that this conclusion generally holds as long as the mild assumption for the function form ( f⁢(t)≥0 , $f^{\prime }(t)>0$ and ${\left(\log f\,\left(t\right)\right)}^{\prime \prime }\leq 0$ ) is satisfied. In the electronic supplementary material, appendix S1.1, we provide a quantitative result for a specific case where f⁢(t) is linear. Note also that in the repaid condition, $t_{p}^{*}=T$ if the intersection of the two curves in figure 1 is in the unfeasible range (T,2⁢T) (figure 1*b*). In this case, the first player invests all available time in exploration, while the second player will choose *t*_*s*_ such that $T+t_{s}$ corresponds to the intersection of the two curves. As shown in the electronic supplementary material, appendix S1.1, this occurs (i.e. $t_{p}^{*}=T$ and $t_{s}^{*}>0$ ) only if function f⁢(t) is convex. By contrast, if $t_{p}^{*}<T$ (this holds whenever f⁢(t) is linear or concave), the second player would invest all available time in exploitation ( $t_{s}^{*}=0$ ) (figure 1*a*).

The above formal model predicts that for a fairly broad class of learning curves ( f≥0 , $f^{\prime }>0$ and ${\left(\log f\right)}^{\prime \prime }\leq 0$ ) rational players would invest more in exploration in the repaid condition than in the unrepaid condition. In §3, we investigate whether this prediction of a simple formal model would also apply to artificial agents working on the virtual arrowhead task by means of computer simulations.

## 3. Computer simulations

We conducted computer simulations in which hypothetical agents equipped with a seemingly rational learning algorithm work on the same virtual arrowhead task as in the laboratory experiment to investigate if such agents invest more in exploration in the repaid than in the unrepaid or asocial condition as predicted by the formal model.

**Figure 2.**
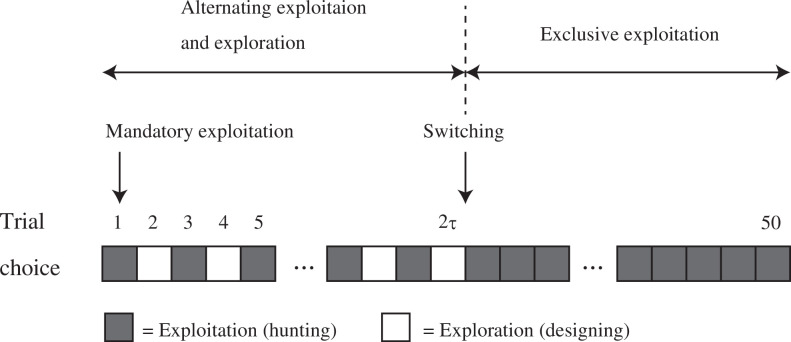
The time-allocation strategy of agents assumed in computer simulations.

In previous studies using the virtual arrowhead task, it has been assumed that an agent could engage in both exploration (designing an arrowhead) and exploitation (going on a hunt) in a single trial. By contrast, in the computer simulations and laboratory experiment of the present study, agents or participants had to choose either exploration or exploitation in each trial. Given that each agent or participant is endowed with T=50 trials and forced to choose exploitation in the first trial, there are $2^{T-1}=2^{49}$ possible allocation strategies. Since it is not practical to search the entire space of all possible allocation strategies, which is vast, we restricted the search space to a specific type of allocation strategy analogous to the optimal strategy found in the formal model. That is, we assumed that an agent engaged in exploitation and exploration alternately from trial 1 to trial 2⁢τ (remember that the first trial is always devoted to exploitation) and engaged only in exploitation from trial 2⁢τ+1 to T , where 0≤2⁢τ≤T-1 (figure 2). In other words, an agent chose exploration in trial 2,4,6,…,2⁢τ , and chose exploitation in the other trials so that a total of τ and T-τ trials are devoted to exploration and exploitation, respectively. Unlike in the formal model, exploration was always followed by exploitation because agents were not informed about the shape of the fitness landscape and hence had to go hunting at least once to check the performance of a new arrowhead design. More specifically, they had to keep updating the directions of modification based on the comparison of the performance scores obtained in the last two exploitation trials. We systematically searched for the optimal number $\tau_{p}^{*}$ of exploration trials that maximized the total pay-off to a first-generation agent in each condition. Note that $\tau_{p}^{*}$ is an analogue of $t_{p}^{*}$ in the formal model. We believe that this type of allocation strategy is highly rational and realistic in the sense that humans can easily implement it. Although there might be more complex strategies that could perform better, such as the repetition of three exploration trials followed by two exploitation trials, such strategies would be less intuitive and more cognitively demanding.

Following the method of Mesoudi & O’Brien, we used the ‘win-stay, lose-shift’ strategy for the learning algorithm of agents. Previous studies show that this algorithm, working in a similar fashion to Skinnerian conditioning, closely mimicked the behaviour of real participants in the virtual arrowhead experiments [26,28]. Each agent had four memory slots, where one was to store the score gained in the latest exploitation trial and the other three were to store the sign of the direction (either + or − ) in which each of three arrowhead attributes (i.e. length, width and depth) is to be modified. When an agent explored a new arrowhead design in a trial, it chose one attribute at random and modified its value by L=5 units in the memorized direction ( + or − ). We chose the value of L=5 because it was the median value of the level of modification observed in laboratory experiments by Mesoudi & O’Brien and Nakawake & Kobayashi [28]. When an agent chose exploitation (i.e. went hunting) in a trial, it received a reward, whose amount depended on the arrowhead design in the same way as in the laboratory experiment (see §4.1). If the reward was less valuable than that of the latest exploitation trial, the agent reversed the direction of modification (e.g. from + to − ) for the attribute modified in the latest exploration trial (lose-shift); otherwise, the direction was left unchanged (win-stay). The directions of modification were also left unchanged for attributes that were not modified in the latest exploration trial irrespective of the score gained through exploitation.

To find $\tau_{p}^{*}$ for each condition, we must describe the total pay-off to a first-generation agent with strategy $\tau_{p}$ in each condition. Let g⁢(τ) denote the mean efficiency (i.e. expected performance score gained from one exploitation trial excluding noise) of arrowhead designs obtained after τ exploration trials, where τ is an integer in the range 0≤2⁢τ≤T-1 . Note that similar to f⁢(t) in the formal model, g⁢(τ) can be interpreted as the learning curve of an average agent. Given that the initial value of g⁢(τ) is g⁢(0) and the final value is $g\,\left(\tau_{p}\right)$ , the expected total pay-off to a first-generation agent with allocation strategy $\tau_{p}$ in the unrepaid condition is given by


(3.1) $$W_{p,u}=\sum_{\tau =0}^{\tau_{p}-1}g(\tau )+(T-2\tau_{p})g(\tau_{p}),$$

where the first term is attributed to the period of alternating exploitation and exploration (trial 1 to $2\,\tau_{p}$ ) and the second term is the period of exclusive exploitation (trial $2\,\tau_{p}+1$ to T ). Note that the first term disappears when $\tau_{p}=0$ (no exploration). The pay-off function $W_{p,u}$ is obtained once the learning curve g⁢(τ) is known. Hence, we first computed g⁢(τ) with agent-based simulations and then computed $W_{p,u}$ using equation (3.1).

Although the repaid condition involves a sequential game of two players, we may assume that the first-generation player has control over both $\tau_{p}$ and $\tau_{s}$ , the strategies of the first- and second-generation players, respectively, for the same reason as in the formal model. Therefore, the total pay-off to a first-generation agent in the repaid condition is given by replacing T in equation (3.1) by 2⁢T :


(3.2) $$W_{p,r}=\sum_{\tau =0}^{\tau_{p}-1}g(\tau )+(2T-2\tau_{p})g(\tau_{p}).$$

**Figure 3.**
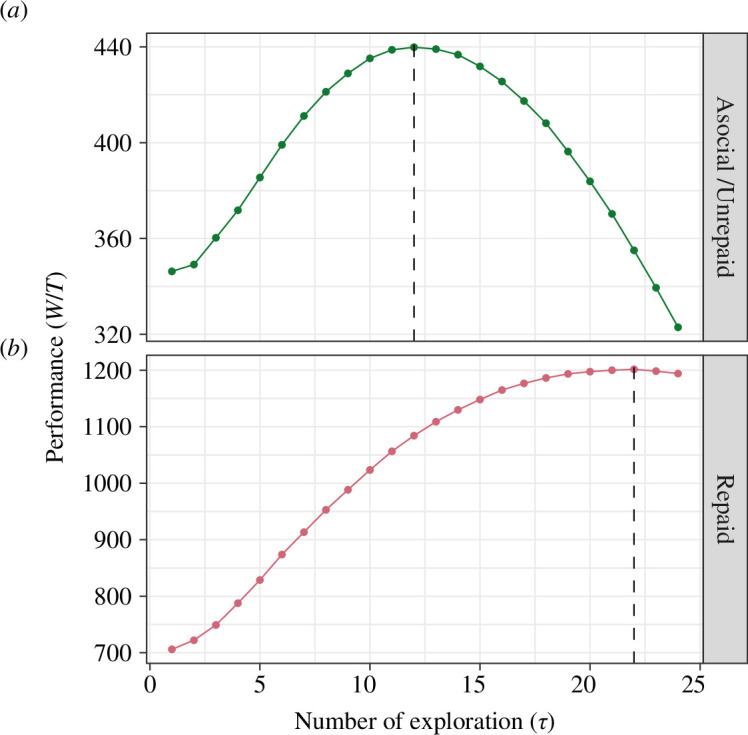
The mean per-trial performance scores in the unrepaid condition $W_{p,u}/T$ (*a*), and in the repaid condition $W_{p,r}/T$ (*b*); both computed from 10 000 runs of computer simulations.

We conducted simulations with 10 000 agents to calculate the learning curve g⁢(τ) as a function of the number τ of exploration trials under the assumption of alternating exploitation and exploration; the resulting learning-curve function was then used to obtain pay-off functions $W_{p,u}$ and $W_{p,r}$ in equation (3.1) and equation (3.2), respectively. To find the optimal number $\tau_{p}^{*}$ of exploration trials for the first-generation agents, we plotted the per-trial performance scores ( $W_{p,r}/T$ and $W_{p,u}/T$ ) as functions of $\tau_{p}$ in both repaid and asocial/unrepaid conditions (figure 3). As the figure shows, the optimal number of exploration trials is $\tau_{p}^{*}=22$ for the repaid condition and $\tau_{p}^{*}=12$ for the unrepaid or asocial condition. This implies that rational agents should terminate exploration as early as trial 24 , approximately half of the total available time ( T=50 ), in the unrepaid or asocial condition while they should alternate exploitation and exploration up until trial 44 , almost the end of the given time, in the repaid condition. Thus, agents should invest considerably more time in exploration in the repaid condition than in the unrepaid or asocial condition.

To confirm the generality of the above result, we also conducted the same analyses using other learning curves, which were derived from the laboratory experiments and computer simulations of our previous study and based on different set-ups (e.g. fitness landscapes). The results again show that agents should invest more in exploration in the repaid condition than in the unrepaid or asocial condition (see the electronic supplementary material, figures S3 and S4).

## 4. Laboratory experiment

### 4.1. Experimental method

#### 4.1.1. Participants

A total of 100 students (undergraduates and graduates, aged 18–27 years; gender: 44 females, 54 males and two other) in Kochi Prefecture participated. The sample size was determined based on the capacity of our participant pool (the experiment was conducted in 2022, in which the pool was limited owing to the Covid pandemic). Each participant was randomly assigned to one of three conditions: 20 participants were assigned to the asocial condition, 40 participants (20 pairs) were assigned to the repaid condition and 40 participants (20 pairs) were assigned to the unrepaid condition. All participants were recruited from the participant pool of Kochi University of Technology. Each participant received a show-up fee of 700 Japanese yen (JPY) and an additional payment whose amount depended on the participant’s performance in the experiment. The resulting total payments to the individual participants ranged from 1320 to 3340 JPY (see §4.1.3). The show-up fee and the rate of conversion between performance and the additional part of the payment were determined so that the total payment to each participant hardly (practically never) falls short of the corresponding legal minimum wage of Kochi Prefecture at that time ( ≥ 820 JPY h^-1^) and this account was approved by the ethical committee of Kochi University of Technology. All participants completed a consensus form before starting the experimental task.

#### 4.1.2. Design

We set three experimental conditions: an asocial condition as the baseline treatment, and two social conditions, that is, the repaid and unrepaid conditions. In the asocial condition, there is no transmission of arrowhead designs between participants, while there is such transmission in the repaid and unrepaid conditions. For the repaid and unrepaid conditions, we adopted the transmission-chain method [29,31–33] with two generations. Namely, participants were randomly paired and acted as the first or second generation of a chain, where the final arrowhead design of the first generation was used as the initial arrowhead design for the second generation. The repaid and unrepaid conditions were equivalent in all respects except with regard to pay-offs to the first-generation participants. In the unrepaid condition, participants in the first generation received only the payment based on their own scores as in the asocial condition. On the other hand, in the repaid condition, participants in the first generation received an additional payment, which was equivalent to the variable part (i.e. not including the show-up fee) of the payment to their second-generation partner. In both repaid and unrepaid conditions, payments to the second-generation participants depended only on their own scores as in the asocial condition.

#### 4.1.3. Experimental task

The experiment was conducted in the experimental laboratories of Kochi University of Technology, where the anonymity of participants was preserved. For the experimental task, we built an executable program with Visual Basic (Visual Studio 2019) by modifying that used in a previous study [28], which reimplemented the original arrowhead task of Mesoudi & O’Brien [26] and is publicly available at GitHub (https://github.com/YNakawake/projectile_neg). Participants also answered a prequestionnaire and postquestionnaire, which were both paper based. The entire procedure of the experiment is described in the electronic supplementary material, appendix S2.1 and the details of experimental materials are described in the electronic supplementary material, appendix S2.2.

The experimental task for each participant consisted of 50 trials, which were called ‘days’ during the experiment. On each day or trial, participants chose one of two actions: designing a new virtual arrowhead (exploration) or going on a hunt with the arrowhead on hand (exploitation). The first day was an exception, however, as hunting was the only available choice, and hence, each participant chose between the two actions 49 times in total. The screen displayed to the participants consisted of two panels, a main panel and a side panel on the right side. In the side panel, two large buttons corresponding to exploration and exploitation were arranged vertically and labelled ‘design the arrowhead’ and ‘go to the hunting ground’, respectively (see the electronic supplementary material, figure S6*a*). Once a choice was made between the two actions by clicking either button, the main panel displayed content specific to the chosen action.

When participants chose ‘design the arrowhead’, they were allowed to modify one of the three attributes of the arrowhead (length, width and thickness) by up to five units. Each attribute was allowed to take integer values of between 1 and 100 so that participants could not choose values outside of this range (see the electronic supplementary material, figure S6*b*). After changing the value of one attribute, participants proceeded to the next step by clicking a button reading ’make the arrowhead into this shape’. Once it was clicked, an image of flintknapping appeared. If the length or width was changed, the picture of the arrowhead displayed in the main panel also changed in proportion to the chosen values (we did not visualize the thickness of the arrowhead following previous studies; [28]). The initial value for each attribute was fixed and common for all participants except the second-generation players in the repaid and unrepaid conditions, for whom the initial value was the final value of the first-generation counterpart.

When participants chose ‘go to the hunting ground’, their score was displayed on the screen as calories acquired from hunting. The score ranged from 0 to 1000 and was computed from the current arrowhead design and the fitness landscape predefined on the three-dimensional space of the arrowhead attributes (see the electronic supplementary material, figure S6*c*). We used a unimodal fitness landscape with a quadratic function (see the electronic supplementary material appendix S2.3) following a previous study using the virtual arrowhead task [29]. The value calculated from the fitness landscape was the expected score for the current arrowhead design, and random noise ( $\varepsilon ∼N\,\left(0,5^{2}\right)$ ) was added to it to yield the actual score displayed to the participant. The score was shown by a numeral as well as a bar indicator with an animal pattern. The score gained in the last hunting trip was always displayed on the screen by a numeral so that participants could compare the current and previous scores when they went hunting. The score of the last hunting trip was updated only when participants returned from a hunting trip. Therefore, the participants had to go hunting at least once to check the performance of a new arrowhead design. On the screen, the number of days left, the current cumulative score in units of calories, and the amount of payment in JPY equivalent to the current cumulative score (not including the show-up fee) were also displayed during the experiment. At the end of the experiment, participants received a payment in JPY based on their cumulative score W . The payment was given by 2⁢W/50 rounded up to the nearest 10 plus 700 for the show-up fee. In addition, the first-generation participants in the repaid condition received an additional payment as explained in §4.1.2.

### 4.2. Results

#### 4.2.1. Selection of exploration or exploitation

As we assumed in the computer simulations, participants tended to alternate between exploration and exploitation until a certain point and then to switch to exclusive investment in exploitation (see the electronic supplementary material, appendix S2.4). In addition, participants’ responses on the postquestionnaire were consistent with this pattern (electronic supplementary material, appendix S2.4).

**Figure 4.**
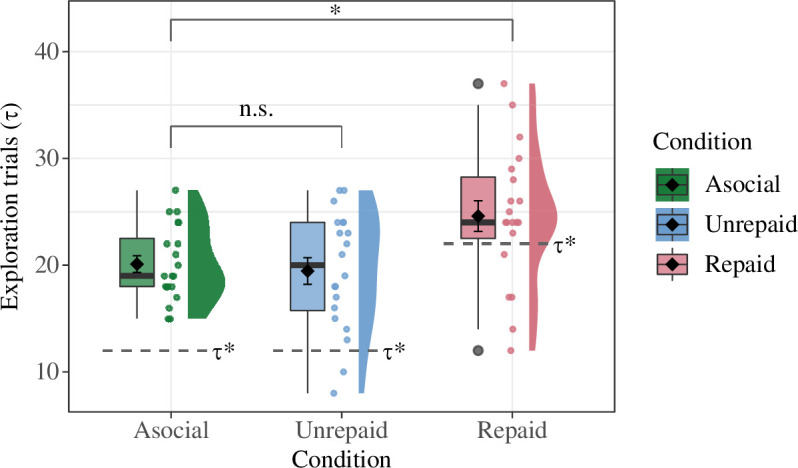
The number of exploration trials observed among first-generation participants in the asocial (left), unrepaid (centre) and repaid (right) conditions. The diamond and error bars in each box plot represent the mean and standard error of the 20 participants in the corresponding condition, while the box ranges from the first to third quantile and the thick horizontal bar represents the median. Dots represent the results for individual participants, which are horizontally perturbed for visibility. On the right side of the dots are smoothed distributions of the individual results. Dashed lines show the optimal number of exploration trials $\tau_{p}^{*}$ obtained through computer simulations. ${\;}^{*}p<0.025$ . n.s. p≥0.025 .

As stated in §1, the aims of the present experiment were to test our hypotheses (i) and (ii). To achieve this goal, we compared the mean numbers of exploration trials ( τ ) in the unrepaid and repaid conditions against those of the asocial condition (figure 4). Since we conducted two t -tests, the significance criteria were adjusted using the Bonferroni correction method ( α=0.025 ); note, however, that we obtained the same result even under the original significance level ( α=0.05 ).

The mean number of exploration trials is significantly higher for the first generation of the repaid condition than for the asocial condition ( $M_{repaid}=24.60$ , s.d.=5.56 , $M_{asocial}=20.10$ , s.d.=3.51 , $t_{29.40}=2.75$ , p=0.010 , d=0.89 ). By contrast, it is not significantly higher for the first generation of the unrepaid condition than for the asocial condition ( $M_{nonrepaid}=19.45$ , s.d.=6.42 , $M_{asocial}=20.10$ , s.d.=3.51 , $t_{32.06}=-0.44$ , p=0.661 , d=-0.14 ). The small Cohen’s d ( d=-0.14 ) value suggests that the effect, if existent, was negligible and that the sign of the effect size was the opposite to that expected under hypothesis (i). To summarize, hypothesis (ii) was supported and hypothesis (i) was rejected. Thus, the results suggest that the presence of the future generation does not increase investment in exploration by participants without the repayment mechanism, as predicted by the theory of rational agents. We also conducted some analyses on the number of times exploration was chosen by the second-generation participants, the results of which are given in the electronic supplementary material, appendix S2.5.

To exclude the possibility that a chance variation in participants’ prosociality between conditions caused or affected the above result, we measured the prosociality of each participant (i.e. social value orientation; [36]) through the postquestionnaire. We conducted regression analyses using the number of exploration trials as the dependent variable and experimental conditions and the prosociality of participants as independent variables (see the electronic supplementary material, appendix S2.6). The results of the regression analyses showed that the above conclusion was unchanged (i.e. hypotheses (i) and (ii) are rejected and supported, respectively) while prosociality did not have a significant effect on the number of exploration trials. Thus, prosociality is unlikely to be a cause of the observed difference in the number of exploration trials between conditions.

Note that participants in general chose exploration more frequently than expected from the optimal switching point $\tau_{p}^{*}$ found in the computer simulations (figure 4). This tendency towards over-exploration was especially remarkable in the asocial and unrepaid conditions. Note that the learning algorithm of the agents of the computer simulations (i.e. ‘win-stay, lose-shift’ strategy) took into account uncertainty about the fitness landscape and random noise. However, the optimal switching point $\tau_{p}^{*}$ was derived from the comparison of the expected pay-offs for various $\tau_{p}$ -values, which were information never available to each participant in the laboratory experiment. The tendency towards over-exploration found in the experiment might be attributable to this lack of information, that is, participants needed to ‘waste’ some trials to notice that they had already passed the optimal switching point (not the optimal design), or in other words, the marginal benefit of exploration had already become lower than that of exploitation. Another factor that possibly contributed to the discrepancy between the simulations and experiment may be our limited assumption on the allocation strategy of the agents in the simulations. Namely, we assumed that the agents alternate exploration and exploitation up to the time of switching but some of the participants in the experiment deviated from this pattern (electronic supplementary material, appendix S2.4). Perhaps less important than those factors, curiosity might also have played a role in postponing the switch.

#### 4.2.2. Efficiency

**Figure 5.**
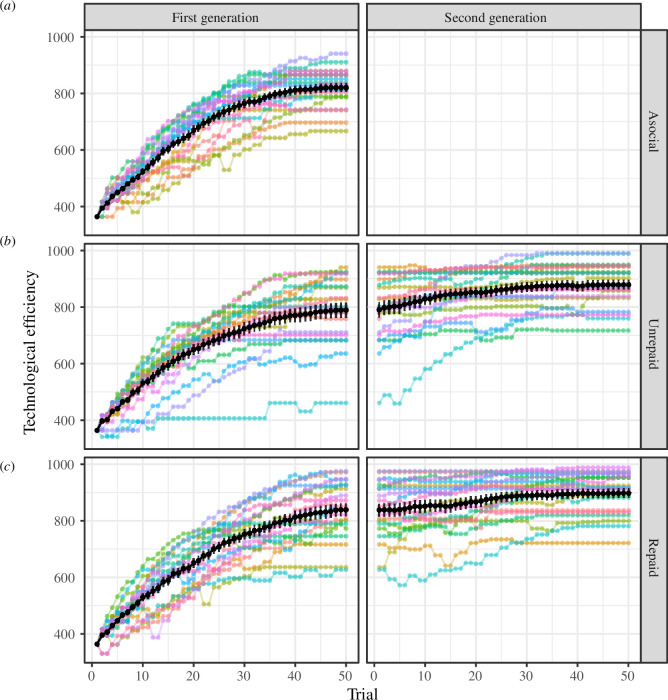
The dynamics of the efficiency (the performance score excluding noise) of each participant’s arrowhead across 50 trials in the asocial (*a*), unrepaid (*b*) and repaid (*c*) conditions. The left and right columns represent the results of the first and second generations, respectively, and lines denote the results of individual participants. Note that lines are flat where participants chose hunting and hence their arrowhead designs did not change. Black lines show the mean over the 20 participants in each generation in each condition, where error bars show the standard errors.

The dynamics of the efficiency (i.e. the performance score before random noise was added) of each participant’s arrowhead over the 50 trials are visualized in figure 5. As the figure shows, the efficiencies of the arrowheads showed an overall tendency to increase with the number of trials across the two generations. The figure also suggests that the presence of a succeeding generation (i.e. the first generation of the repaid and unrepaid conditions as opposed to the asocial condition) resulted in little or no improvement in the efficiencies of the final arrowhead designs. We conducted t -tests and confirmed that there were no statistically significant differences in the mean efficiency levels of the final arrowheads between the repaid and asocial conditions ( $M_{repaid}=838.70$ , s.d.=103.16 , $M_{asocial}=820.25$ , p=0.511 , d=0.21 ) and between the unrepaid and asocial conditions ( $M_{unrepaid}=789.90$ , s.d.=119.59 , $M_{asocial}=820.25$ , s.d.=69.02 , $t_{30.39}=-0.982$ , p=0.333 , d=-0.31 ).

As we mentioned in §4.2.1, participants in the first generation of the repaid condition invested more in exploration than those in the asocial condition, but those in the first generation of the unrepaid condition did not. Therefore, one might expect that at least the repaid condition must have achieved a higher average level of efficiency than the asocial condition at the end of the first-generation stage; however, this was not the case. There are two possible reasons for this somewhat counterintuitive result. First, the variance in efficiency between individuals might have been too large to detect the difference between the conditions. Second, the searching behaviour of the participants in the repaid condition may have been overly exploratory relative to the learning algorithm in computer simulations, which is relatively conservative. For the width, for example, approximately 40% of participants in the first generation of the repaid condition *overshot* the optimal attribute value, while this occurred only among 15% and 10% of participants in the asocial condition and the first-generation stage of the unrepaid condition, respectively (see the electronic supplementary material, figure S5).

## 5. Discussion

### 5.1. Summary of results

The present study investigated the exploration–exploitation trade-off, which is inherent to the cumulative cultural evolution of technology, in an intergenerational framework. We formulated two hypotheses: (i) exploration of technology is promoted solely by the fact that technology is transmitted to future generations; and (ii) it is promoted if individuals are rewarded for providing increased benefits to future generations. We used three approaches to test the hypotheses: a formal model, computer simulations and a laboratory experiment. In the formal model, which ignores informational uncertainty as well as behavioural irrationality, we were able to develop an appropriate rewarding system (i.e. the repaid condition) and showed that hypothesis (ii) holds true for a broad class of learning-curve functions under the proposed rewarding system. The computer simulations confirm that rational agents again followed hypothesis (ii) under the same reward system even in the set-up of the laboratory experiment, which incorporated informational uncertainty. Finally, the laboratory experiment, in which participants were not necessarily rational, supported hypothesis (ii) under the same reward system used in the former two approaches. However, the experiment did not support hypothesis (i). In summary, our results supported hypothesis (ii) but not (i), as predicted by the theory of rational players (i.e. personal pay-off maximization).

The above results are consistent with the findings of previous studies on intergenerational dilemmas whereby cooperation with future generations is unlikely to be robustly sustained unless additional mechanisms are added [19,20,22]. The mechanism introduced in the present study is a system that incentivizes individuals to increase investments in exploration by rewarding them for the success of the next generation, that is, the successors of their technologies. Alternatively, psychological intervention (e.g. taking the perspective of future generations or activating certain emotions) might promote technological exploration [22,23]; however, altruistic actions induced by such emotion-based treatments might be less robust than ‘cooperative’ actions sustained by incentive-based mechanisms such as ours [21].

### 5.2. Implications of the negative result

Our experiment could not demonstrate that humans are willing to develop new technologies at the expense of their own benefit for anonymous strangers from future generations (i.e. hypothesis (i) was rejected). This is not a trivial result for us for the following reason. If natural selection overall tends to favour an increased effort for exploration in the presence of successors of technology in the field, we may expect that humans have evolved a general tendency to increase the investment in exploration when they meet some successors of their technologies. If humans have indeed evolved this general tendency, they might also unnecessarily or erroneously express this innate psychological trait even in the unrepaid condition of our laboratory experiment, in which there is no biological reason to do so owing to complete anonymity unlike in the field. This is a hypothesis worth testing given the results of existing experimental studies; for example, humans care about the pay-off to their partner in the Dictator game [37] even in a completely anonymous set-up. Thus, our experiment might have potentially contributed an indirect evidence of the hypothetical selection pressures in the field if the general tendency in question had been detected.

However, we could not detect such a general tendency in the unrepaid condition. This allows at least two interpretations: (i) one is that humans have not evolved it but rather evolved to adjust the degree of exploration flexibly and finely in response to the benefits and costs of increased exploration in each specific situation, as the participants did in the repaid condition; and (ii) another is that the general tendency exists but our unrepaid set-up lacked some key factors to elicit it. Hereafter, we take the standpoint of the first interpretation for a while but later come back to the second interpretation to discuss possible future extensions of the unrepaid set-up (§5.5).

According to the first interpretation, whenever people appear to make overly large investments in technological exploration, there should exist selfish or genetic returns that are worth the cost. Such returns may come in a variety of forms in the short or long term: monetary or material returns from selling or trading innovative products, an increased survival rate for one’s own group in competition with other groups, prestige or a positive reputation in society [38,39], sexual appeals to potential reproductive mates [40–43] or indirect benefits through the success of genetically related individuals (e.g. offspring or younger siblings; [24,44]). In traditional societies in which most subsistence skills are transmitted from parents or genetically related elders [45–47], there seems considerable room for the evolution of over-exploration to benefit genetic relatives, although evidence would hardly be available. Needless to say, research and design (R&D) activities of modern enterprises are mainly aimed at monetary returns rather than at the personal use of technologies.

### 5.3. Implications for costly teaching

Similar arguments to the previous subsection might apply to the evolution of costly teaching [48–50], that is, humans might not enjoy transmitting technologies to anonymous strangers at a cost. If this is the case, costly teaching with no direct return must occur only between genetically related individuals, and in all other cases, teaching should accompany net selfish benefits. Recently, Ventura & Akcay [51] have developed a cognitive-evolutionary model of teaching, in which the objective function of a teacher (i.e. first generation) is given by the sum of (i) its own direct fitness (including an energetic cost of teaching), and (ii) the fitness of the naive learner (i.e. second generation) weighted by the relatedness r between the teacher and learner. In their model, increasing the learning efficiency comes with an opportunity (not energetic) cost of a decrease in the direct fitness. As expected, their result shows that active teaching effort evolves only if the relatedness r is sufficiently large.

Ventura and Akcay’s result fully conforms with our model’s prediction that an increased effort for exploration to benefit the second generation comes with an opportunity cost and hence can be favoured only in the repaid condition. In future theoretical and experimental work, it may be interesting to combine the two kinds of opportunity costs (i.e. those of exploration and teaching) into a single framework. For example, we may assume that the transmission of the arrowhead design between the two generations comes with some error and the first-generation participant can choose the third behavioural option (say, ‘teach’) to increase the faithfulness of transmission. We expect that the transmission error and the additional opportunity cost owing to teaching both make the extended exploration to benefit the second generation less effective and hence less attractive; therefore, the optimal investment in exploration by the first generation would probably decrease. Yet another interesting extension may be to impose an opportunity cost of teaching on the side of the second generation as well, as in Nakata & Takezawa’s model [52], which further complicates the decision making by the first generation.

### 5.4. Implications for evolutionary dynamics

Our models (the formal model and simulations) are designed to agree with the design of the simple laboratory experiment with only two generations. As an inevitable consequence, they have many restrictive assumptions, which make direct comparison difficult between the model and cultural evolution in the field. One of the serious limitations in this respect is that in the repaid set-up of our models, the reward for benefitting the successor of technology is taken as given. Although we already made verbal arguments to justify this set-up, more formal justifications through evolutionary models should be desirable. As to kinship as a mechanism to generate the reward (i.e. an indirect fitness or genetic benefit), Kobayashi *et al.*’s gene-culture coevolution model [24], which is based on population genetics and evolutionary game theory, might fit this purpose. In their model, juveniles socially learn cumulatively evolving technology from their own parents with a certain probability q and from unrelated elders with probability 1-q . They assume that investments in social/individual learning as well as exploitation (in the presence of a time-allocation trade-off) coevolve with the level of technology. They find, as expected, the evolutionarily stable investments in learning and the equilibrium level of technology both increase with the vertical transmission rate q . This result seems to be consistent with the optimal behaviour of the first generation in our models (see also Ohtsuki *et al.* [53]).

Apart from kinship, there are several other mechanisms through which an increased investment in exploration is rewarded (e.g. increased prestige or sexual appeal), as argued earlier. We do not know of any gene-culture coevolution model of cumulatively evolving technology that explicitly deals with these mechanisms, and hence it is worth developing new models in future work. Those models would be very different from each other, depending on what specific mechanism is adopted. For example, as to the benefit through increased prestige or social status [38,39], the model must explain the selection pressure for a societal mechanism to confer higher prestige to skilful individuals and bring extra benefits to the prestigious people. A plausible assumption would be that skilful individuals can control the spread of their skills and this creates a selection pressure for the societal system to exchange access to resources (e.g. food or mating opportunities) for experts’ valuable knowledge or its products [39]. The benefit through increased sexual appeal [40–43] can be even more challenging to model because it involves sexual selection; that is, we need to work on an explicit two-sex model in which time-allocation strategies and cumulative technology evolve on one sex and mate choice preference on the other, although it is also possible that technology is transmitted bilaterally and both sexes evaluate the competency of each other.

### 5.5. Miscellaneous arguments on future extensions

So far we have been assuming that humans have not evolved an innate tendency to extend exploration in response to the presence of successors. Now, let us take another standpoint that humans have such an innate tendency but our unrepaid set-up lacked some key factors to elicit it. It must be noted that in many realistic situations where cultural transmission occurs, individuals may not be complete strangers as they were in our experiment. Therefore, participants may be more willing to explore new technologies for later generations even without monetary repayment if anonymity is moderated or interactions between adjacent generations, for example, one- or two-way communication via text or other kinds of messages, are allowed. For example, we may allow the second generation to rate the inherited arrowhead design with a numerical score, which is then displayed to the first generation at the end of the experiment (of course, the first generation must be informed of this treatment ahead of time). We may also allow the second generation to send a simple one-line message such as ‘thanks in advance’ to the first generation at the beginning of the experiment, which might affect the behaviour of the first generation. The unrepaid condition in the present study will be a baseline for comparison in these future experiments with moderated anonymity.

One limitation of our experiment is that it used only a specific fitness landscape. Although our formal model and computer simulations enable us to extrapolate the results of the experiment to a broad class of fitness landscapes, they are still confined to smooth and unimodal landscapes. In multimodal landscapes, the condition whereby the learning-curve function is monotonically increasing ( $f^{\prime }>0$ ) may be violated since the efficiency of the technology must temporarily decrease to travel across a fitness valley [26]. Nevertheless, if human technologies tend to improve with the amount of effort invested in exploration in the long term, though the improvement is occasionally punctuated by fitness valleys, we believe that sufficiently far-sighted agents are not necessarily bothered by a short-term decrease in f and hence similar conclusions may be drawn. Further research is, however, required for more rigorous arguments on multimodal landscapes.

Another limitation is that arrowhead designs were transmitted only between two generations, while past experimental studies have often allowed transmission over three or more generations [31,33]. Previous theoretical studies on cultural evolution have often been motivated by free-rider problems, where social learners were viewed as scroungers of information produced by individual learners [14,15,24]. In a transmission-chain framework such as ours, if a first-generation individual makes an over-investment in exploration for later generations but the second-generation individual does not care about the third or later generations, the second-generation individual should be regarded as a free-rider who only receives the benefit of altruism and does not reciprocate it. Therefore, we must extend the experiment to allow transmission over at least three generations to study free-rider problems. Such an extension would allow us to relate the cultural transmission experiments to experiments on free-riders in non-cultural frameworks, for example, those on intergenerational social dilemmas [19,20,22] or pay-it-forward reciprocity [18].

Our simulation model with the simple win-stay learning algorithm, which borrows from Mesoudi & O’Brien’s [27] experimental study on cultural evolution, successfully predicted the behaviour of real participants at least at a qualitative level, suggesting the potential usefulness of *in silico* experiments in the analysis of experimental data. Obviously, however, this means neither that the present model is true nor that it is the best model in predictability. Further, the model does not explain the causes of the behavioural variations between individuals in the data. Thus, there is much room to improve this approach. In future work, it would be more desirable to incorporate psychological parameters, which allow explaining behavioural variations (e.g. the probability that a ‘loser’ changes the direction of modification for an arrowhead attribute). Combined with data, such a model would allow estimating the parameter values for each participant and hence understanding the process of decision making on an individual scale. It would also allow comparing different models in terms of predictability or plausibility using information criteria. We consider that such intensive use of models and simulations is a promising future direction for the field of cultural evolution experiments.

In this article, one’s own cumulative score and that of the partner are equally weighted in the pay-off function of the first-generation player. This assumption was made for analytical simplicity both in the model and experiment. In realistic economic contexts, these may be differently weighted, where one’s own score should often be given a higher weight. In biological contexts, on the other hand, the parent’s score may affect the number of offspring, to which the technology should be transmitted, as in previous gene-culture coevolution models of cumulatively evolving technology [24]. This makes biologically appropriate weighting of the scores of the parent and offspring (i.e. a precise measure of the genetic success of the parent) very complicated, where the weight on the offspring’s score may depend on the score of the parent. In this article, we have considered the case of equal weights as the first step towards these more complex extensions. In future work, different pay-off functions in the repaid condition are worth exploring both theoretically and empirically.

### 5.6. Implications for collective problem solving

We remark that our results may be interpreted in terms of collective problem solving in management [54] or behavioural science [55]. In management science, the ability of organizations to achieve exploration and exploitation simultaneously has been termed ‘ambidexterity’ [56]. It has been argued that ambidexterity can be realized through the division of labour among agents (individuals or organizations) [57]. For example, an organization may focus on R&D and exchange novel technologies for the profits earned by another organization, which focuses on exploitation [54]. The present study is perhaps a novel contribution to this field of research in that it uses the method of a laboratory experiment as well as a computer-based task of cumulatively evolving virtual technology. Specifically, the result of our repaid condition suggests that the division of labour between two individuals is promoted if the profits earned by the specialized exploiter are partly transferred to the specialized explorer. Such a transfer of profits naturally occurs when the two individuals are members of the same household, and it is indeed known that ambidexterity is higher in family-operated firms [9,10].

Behavioural scientists have also been studying the trade-off between exploration and exploitation from the perspective of collective problem solving and the division of labour [55]. Existing results in this field show that members of a group can potentially make better decisions by combining or aggregating information held by individual members [55,58]. However, given that information is a public good freely shared by the members, inefficiency owing to ‘social loafing’ needs be overcome; that is, each individual tends to invest less effort in exploration than when being alone, free-riding on the information provided by other members [17,59]. This type of social dilemma is also known as Rogers’ paradox [14], and the division of labour through trading is suggested as a possible resolution [8,60]; that is, individuals may be motivated to invest in exploration if they are allowed to charge a fee for exploiting their inventions. In the present framework, information flows unidirectionally from the first to the second generation, which inhibits the first generation from social loafing. Moreover, in the repaid condition, the first generation gets paid a ‘fee’ proportional to the quality of the transmitted technology, so that the first generation has an extra incentive for exploration. Thus, our framework may be regarded as proposing a way of promoting the division of labour and thereby resolving the dilemma of social loafing through a combination of two mechanisms: the unidirectionality of information flow and the information access fee. However, it should again be noted that the fee is not paid by the second generation but by the third party in our experiment, and hence our repaid condition does not exactly correspond to one-to-one trading; it rather fits a situation where information producers get benefits through the community, for example, through prestige or patents. Although we still need to be cautious in interpreting our results in terms of the problem of social loafing for the above reason, it would be interesting to extend our framework to include more than two individuals; we may be able to increase the efficiency of a large group by dividing it into subgroups and controlling the flows of information and profits between the subgroups in an appropriate manner [61,62]. The problem of finding optimal ways of subdivision and controlling the information and profit flows would be an interesting research topic again for management scientists and be fruitful from a practical perspective.

## Data Availability

Data and relevant code for this research work are stored in GitHub (https://github.com/YNakawake/intergen_tradeoff) and have been archived within the Zenodo repository [63]. Electronic supplementary material is available online [64].
